# 3D printed water-soluble scaffolds for rapid production of PDMS micro-fluidic flow chambers

**DOI:** 10.1038/s41598-018-21638-w

**Published:** 2018-02-20

**Authors:** Tobias Dahlberg, Tim Stangner, Hanqing Zhang, Krister Wiklund, Petter Lundberg, Ludvig Edman, Magnus Andersson

**Affiliations:** Department of Physics, Umeå University, 901 87 Umeå, Sweden

## Abstract

We report a novel method for fabrication of three-dimensional (3D) biocompatible micro-fluidic flow chambers in polydimethylsiloxane (PDMS) by 3D-printing water-soluble polyvinyl alcohol (PVA) filaments as master scaffolds. The scaffolds are first embedded in the PDMS and later residue-free dissolved in water leaving an inscription of the scaffolds in the hardened PDMS. We demonstrate the strength of our method using a regular, cheap 3D printer, and evaluate the inscription process and the channels micro-fluidic properties using image analysis and digital holographic microscopy. Furthermore, we provide a protocol that allows for direct printing on coverslips and we show that flow chambers with a channel cross section down to 40 μm × 300 μm can be realized within 60 min. These flow channels are perfectly transparent, biocompatible and can be used for microscopic applications without further treatment. Our proposed protocols facilitate an easy, fast and adaptable production of micro-fluidic channel designs that are cost-effective, do not require specialized training and can be used for a variety of cell and bacterial assays. To help readers reproduce our micro-fluidic devices, we provide: full preparation protocols, 3D-printing CAD files for channel scaffolds and our custom-made molding device, 3D printer build-plate leveling instructions, and G-code.

## Introduction

Lab-on-a-chip (LOC) devices are commonly prototyped using polydimethylsiloxane (PDMS) since PDMS offers many desirable properties: easy usage, cheap production, swift integration of tubing, high light transparency, air-permeability and biocompatibility^1,2^. Traditionally, these devices are produced using soft lithography techniques by creating a silicon master pattern with a negative of the desired micro-fluidic channel^2,3^. During fabrication, PDMS is molded on the silicon master. This molding step transfers the pattern of the master to the elastomer, which can then be peeled of the master and sealed using a glass substrate to create a micro-fluidic device. These methods, however, often require specialized expensive equipment and training, making this method rather inaccessible and slow for LOC device fabrication.

An emerging and more accessible LOC prototyping technique is three-dimensional (3D) printing^4^. 3D-printing offers cost-effective and highly adaptive production of micro-fluidic devices, and with the release of commercial consumer-grade 3D printers new possibilities have evolved^5–9^. The most common, cheapest and simplest type of 3D printers are the Fused Deposit Modeling printers (FDM). These printers work by building up 3D objects by depositing molten plastic layer-by-layer. However, micro-fluidic devices fabricated using FDM printers suffer from many drawbacks such as irregular channel shape and channel surface^6,8^, unreliable channel dimension repeatability and poor optical transparency^6,7^, lowering their potential in microscopic applications significantly. Furthermore, undesirable properties of commonly used thermoplastics such as unknown biocompatibility^10^ and limited air-permeability^6^ are problematic for experiments involving cells or tissue^5,11–14^.

To overcome issues with cell or tissue assays, more sophisticated ways to fabricate micro-fluidic devices using FDM printers have been developed^15–17^. Instead of fabricating the whole micro-fluidic device out of thermoplastic, only the channel scaffold was printed using acrylonitrile butadiene styrene (ABS) plastic^15^ or isomalt^16,17^ as printing material. Subsequently, the printouts were embedded in PDMS or epoxy resin and dissolved using acetone (ABS) or water (isomalt), leaving an imprint of the channel scaffold in the hardened PDMS or epoxy resin. Using this fabrication procedure, micro-fluidic devices with reasonable biocompatibility and air-permeability can be realized. However, problems with poor optical transparency and irregular channel shape remain unsolved. Furthermore, removing the ABS channel scaffold involves strong solvents (acetone) and sugar printers are not commercially available, making them inaccessible for a broad research community.

In this article, we realize biocompatible micro-fluidic devices with predictable shape by embedding a 3D printed water-soluble channel scaffold in PDMS using a regular FDM 3D printer. For that purpose, we provide detailed fabrication protocols for high pressure PDMS flow chambers and PDMS flow chambers on a coverslip. We characterize our micro-fluidic devices by scrutinizing their shape, optical transparency, surface roughness and deviations in physical channel dimensions between CAD design and printout. Furthermore, we show that we can print reproducible channel scaffolds with a cross section of 40 × 300 µm. By experimentally determining the fluid velocity profile inside our flow chamber using digital holographic microscopy, we confirm the predicted micro-fluidic properties of our device. We also provide details about leveling the build-plate of our 3D printer and slicing software settings, information which are rarely published but crucial to reproduce our proposed protocol successfully. Our micro-fluidic devices are cheap, fast to produce, optically transparent, biocompatible, air-permeable, reproducible and tunable, allowing readers to change the channel design to fit their experimental requirements.

## Results and Discussion

### 3D Printer Build-Plate Leveling

To achieve optimal printing accuracy and repeatability, we first level the build-plate of our 3D printer (Ultimaker 2+, Ultimaker, Netherlands). Before starting, we recommend to heat the build-plate to its operating temperature (in this case 60 °C) 60 min prior to the build-plate leveling to ensure thermal equilibrium. Afterwards, we run a custom-written G-code script keeping the build-plate temperature constant and to heat the printing nozzle to 190 °C. Following this step, the script positions the printing nozzle above one of the build-plate adjustment screws and raises the build-plate from its initial to its predefined zero position. Next, we fine-tune the build-plate height by tweaking the adjustment screw until a contact to the nozzle is established. We repeat this procedure for all adjustment screws until the build-plate is leveled. For the final adjustment, we test-print channel scaffolds with defined height and width. Subsequently, we measure the height of the printout with a micro-meter screw gauge (0–25 mm, Helios-Preisser, Germany) and calculate its deviation to the design height. Next, we add this deviation as an offset to the G-code of the printout to compensate remaining leveling errors and repeat the test-printing until we reach an absolute difference in height of ±10%. Our commented calibration G-code can be downloaded from the electronic supplementary information, see the Data Availability section.

### Fabricating High Pressure Resistant PDMS Flow Chambers using 3D Printed Channel Scaffolds

**Figure 1:**
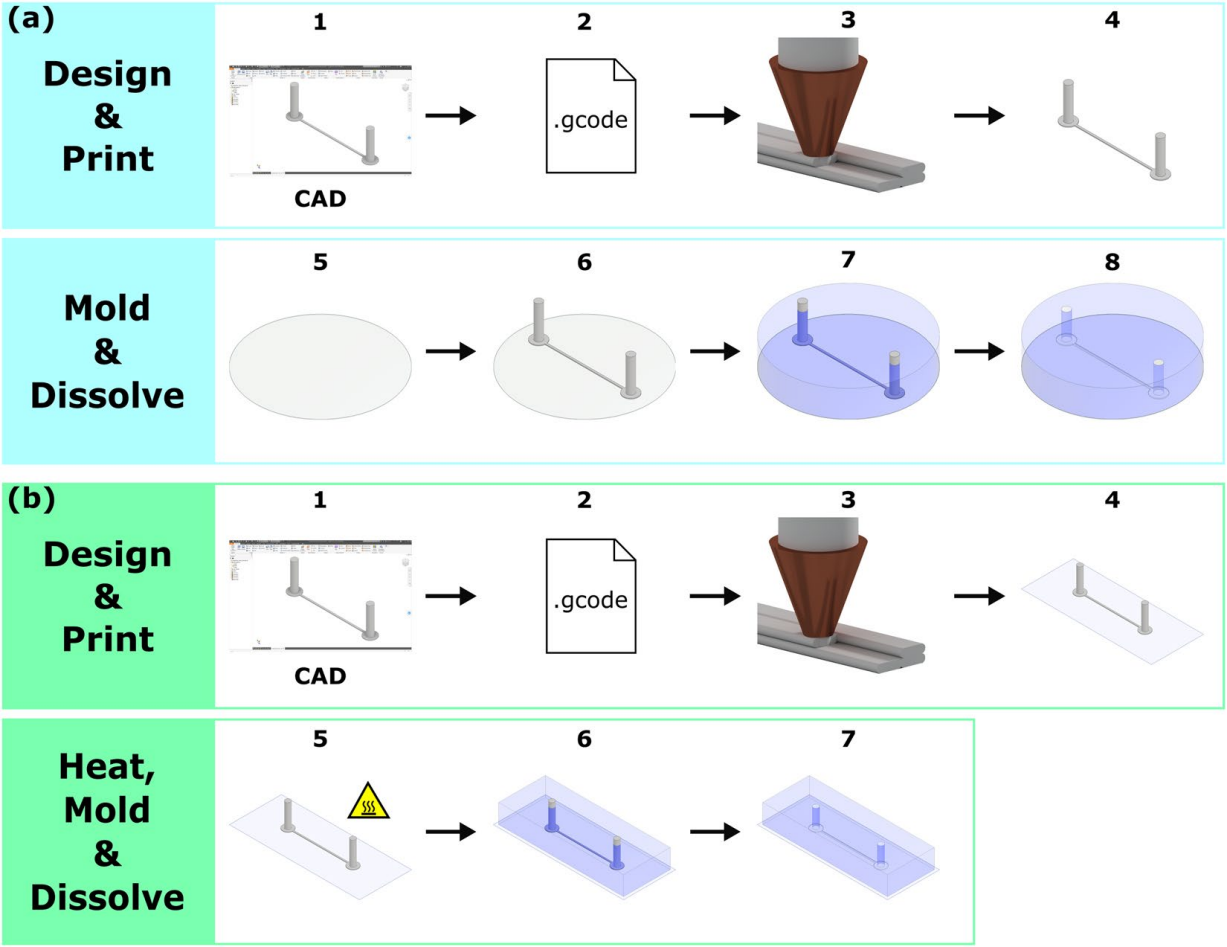
(**a**) Fabrication procedure of high pressure PDMS flow chambers. (1–2) We design a customized channel scaffold using Autodesk Inventor Professional 2017 and translate the scaffold design into G-code using a slicing software. (3–4) We print the channel scaffold using a 3D printer using a water-soluble printing filament. (5–6) After curing a thin layer of PDMS on the bottom of a petri dish, we transfer and center the printed channel scaffold on the former using a pair of tweezers. (7–8) To finish the molding procedure, we pour and cure a second layer of PDMS on top of the scaffold. To dissolve the channel scaffold, we sonicate the flow chamber in a water bath. (**b**) Fabrication procedure of PDMS flow channels on a coverslip. Fabrication step 1–2 is identical to the procedure above. However, in (3–4) we now print the channel scaffold directly on a coverslip. To remove shape irregularities in channel shape, (5) we heat the printout on the coverslip on a hot plate. (6–7) To embed the channel scaffold, we clamp the printed coverslip in a custom-made molding device and add PDMS on top. After curing, we remove the coverslip/scaffold/PDMS assembly from the molding device and dissolve the channel scaffold in an ultrasonic water bath.

To realize high pressure flow chambers, we first design a simple channel scaffold consisting of the actual flow channel connected to an in- and outlet using Autodesk Inventor Professional 2017. Subsequently, we convert the CAD file into 3D printing code using the slicing software Cura 2.6.1 (Fig. 1a step 1 and 2). Next, we print the channel scaffold using an Ultimaker 2+ 3D printer equipped with a 250 µm nozzle and water-soluble, biocompatible polyvinyl alcohol filament (PVA, PrimaPVA 3D Prima) as printing material (Fig. 1a step 3 and 4). Please note, PVA is hygroscopic and longer exposure to the environment causes water uptake. As a result, this water vaporizes during the printing process and changes the size and shape of the printout significantly. Therefore, we recommend to store the filament in a dry and airtight container after usage, preferably with a desiccant. If left mounted on the 3D printer over an extended period in a humid environment the filament can be dried in an oven at 80 °C for 12 hours to restore desired filament properties.

The channel scaffold is printed directly on the pre-heated build-plate of the 3D printer. To ensure optimal printing accuracy, it is important to level the build plate thoroughly. Misalignment or tilt in the build-plate degrades the width and height accuracy and reproducibility of the printout. Besides build-plate leveling, optimized Cura settings such as filament feeding rate and printer nozzle temperature are also crucial to ensure best printing performance. A short summary of Cura settings used in this article can be found in supplementary information (Table S1). The complete Cura profile can be downloaded from electronic supplementary information, see the Data Availability section.

Following the printing step, we inspect the channel scaffold for shape anomalies due to water vaporization or printing artifacts such as irregular filament extrusion using a standard light microscope (MZ6, Leica, Germany). After approving the channel scaffold quality, we mix polydimethylsiloxane (PDMS, Sylgard 184, Dow Corning) base and curing agent at a 10:1 volume ratio. Next, we degas 25 mL PDMS mixture using a desiccator connected to a roughing pump (CIT-Alcatel M2012A) for 30 min. As the first step of the molding procedure, we pour a thin layer of PDMS in a petri dish (Sarstedt AG & Co, Germany, $\oslash$ 35 mm). To obtain a layer height of 500 µm, we place the petri dish on an analytical balance (Mettler, AE 240) and pipette 0.46 g PDMS into it which we then allow to self-level. To cure this initial layer, we incubate the petri dish in an oven for 10 min at 80 °C (Fig. 1a step 5). Next, we center the printed channel scaffold on the cured PDMS layer by applying gentle pressure (Fig. 1a step 6). Subsequently, we pour a second layer of PDMS on this assembly to embed the channel scaffold entirely in PDMS and repeat the degassing procedure (Fig. 1a step 7). In the next step, we cure the second PDMS layer in the oven for 40 min at 80 °C. To dissolve the embedded printed channel scaffold, we sonicate the cured PDMS mold in an ultrasonic water bath (Bandelin Sonorex RK 31) for 2.5 h. Eventually, we flush the flow chamber with clean Milli-Q water and dry it in the oven at 80 °C (Fig. 1a step 8). After connecting tubing to in- and outlet parts, the device is ready to use.

To measure the mechanical resistance of these semi-flexible PDMS devices we apply a constant air pressure to the channel inlet (channel dimensions: 160 µm × 700 µm × 20000 µm, height × width × length) while blocking the outlet. By repeating this experiment for 3 channels with equal size, we find that these devices can withstand a pressure of 7 bar without bursting. For flow channels with the aforementioned dimensions, such pressure corresponds to fluid flow velocities of several m/s.

In summary, the printing and molding procedure takes approximately 4 h for one flow chamber. However, our protocol can also be scaled up for mass production, without increasing fabrication time significantly. For example, 10 flow chambers can be prepared within 5 h. The smallest flow chamber that can be realized with our printer is 40 µm × 300 µm (height × width), but its height and width are scalable to the limits of the printers build volume. As an example, we realized flow chambers over a broad range in height (40–400 µm) and width (300–1000 µm). The data are available in the supplementary information. Also, flow chambers fabricated by this procedure are flexible, robust and can sustain high pressures up to 7 bar, with possible applications in particle^18,19^ and cell^20–22^ separation experiments using micro-fluidic flows. Since the Ultimaker 2+ 3D printer prints sacrificial scaffolds up to several decimeters with mm-resolution, this fabrication protocol can also be used to realize bigger compartments such as biological reaction chambers where electronics need to be integrated.

### Fabricating PDMS Flow Chambers on a Coverslip using 3D Printed Channel Scaffolds

To study cell adhesion and receptor-ligand interactions using optical tweezers or to image single molecules in a microscope, the bottom layer of the used flow chamber must be homogeneous, optically transparent and thin (<170 µm). However, realizing the latter with PDMS is challenging since µm-resolution in height of the initial PDMS layer is difficult to achieve. Thin PDMS layers are also fragile, flexible and the transparency depends on the surface properties of the printed channel scaffold. Based on these reasons, pure PDMS flow chambers are not perfectly suitable when the aforementioned experiments are planned.

To overcome these issues, we propose a protocol to fabricate PDMS flow chambers on a coverslip, guaranteeing a well-defined bottom layer height with high optical transparency. The designing step (Fig. 1b step 1) and the channel scaffold conversion into 3D-printing code using Cura (Fig. 1b step 2) are identical to the protocol proposed in the previous section. However, we now print the channel scaffold directly on top of a standard glass coverslip (No. 1, 60 × 24 mm, FischerScientific, Germany) (Fig. 1b step 3 and 4). To avoid further build-plate leveling, we add an offset corresponding to the thickness of the coverslip in the 3D printing code, leaving all other printer settings unchanged. After printing, we place the coverslip for 5 s on a hotplate (C-MAG HS7 digital, IKA) operating at 230 °C to remove channel irregularities from the printout (Fig. 1b step 5). The heat remelts the plastic, resulting in a smooth channel with a semi-elliptic cross section. However, timing is crucial for the heating process. By over-heating the channel scaffold, PVA changes its appreance from milky to brown. As a result, the PVA becomes brittle and insoluble in water. In contrast, under-heating the channel scaffold will prohibit the plastic to remelt properly leaving the surface rough. After heating, we inspect the channel scaffold for shape anomalies due to water vaporization or printing artifacts such as irregular filament extrusion using a standard light microscope. Subsequently, we place the printed coverslip in a custom-made molding device and add PDMS on top (Fig. 1b step 6). Next, we cure the mold 25 min at 80 °C in an oven and dissolve the scaffold in the hardened PDMS via sonication for 10 min (Fig. 1b step 7).

In summary, the printing and molding procedure takes approximately 1 h for one flow chamber. However, this protocol can also be scaled up for mass production, without increasing fabrication time significantly. For example, 10 flow chambers can be prepared within 2 h and flow chambers fabricated by this procedure are optically transparent and can sustain intermediate pressures, with possible applications in particle tracking, optical tweezers and total internal reflection fluorescence microscope experiments. The smallest flow chamber that can be realized with our printer is 40 µm × 300 µm (height × width), but its height and width are scalable to the limits of the used coverslip and the printers build volume. Furthermore, after dissolving the channel scaffold the PDMS part of the flow chamber can be transferred to a new, for example, surface functionalized coverslip since the cleaned PDMS surface adheres to glass without further treatment.

The engineering drawing and CAD file of our custom-made molding device can be downloaded from the electronic supplementary information, see the Data Availability section. In the same reference, we provide an exemplary G-code file with added offset to correct for the coverslip thickness.

### Impact of Heating the Printed Channel Scaffold on Channel Shape, Optical Transparency and Surface Roughness

**Figure 2:**
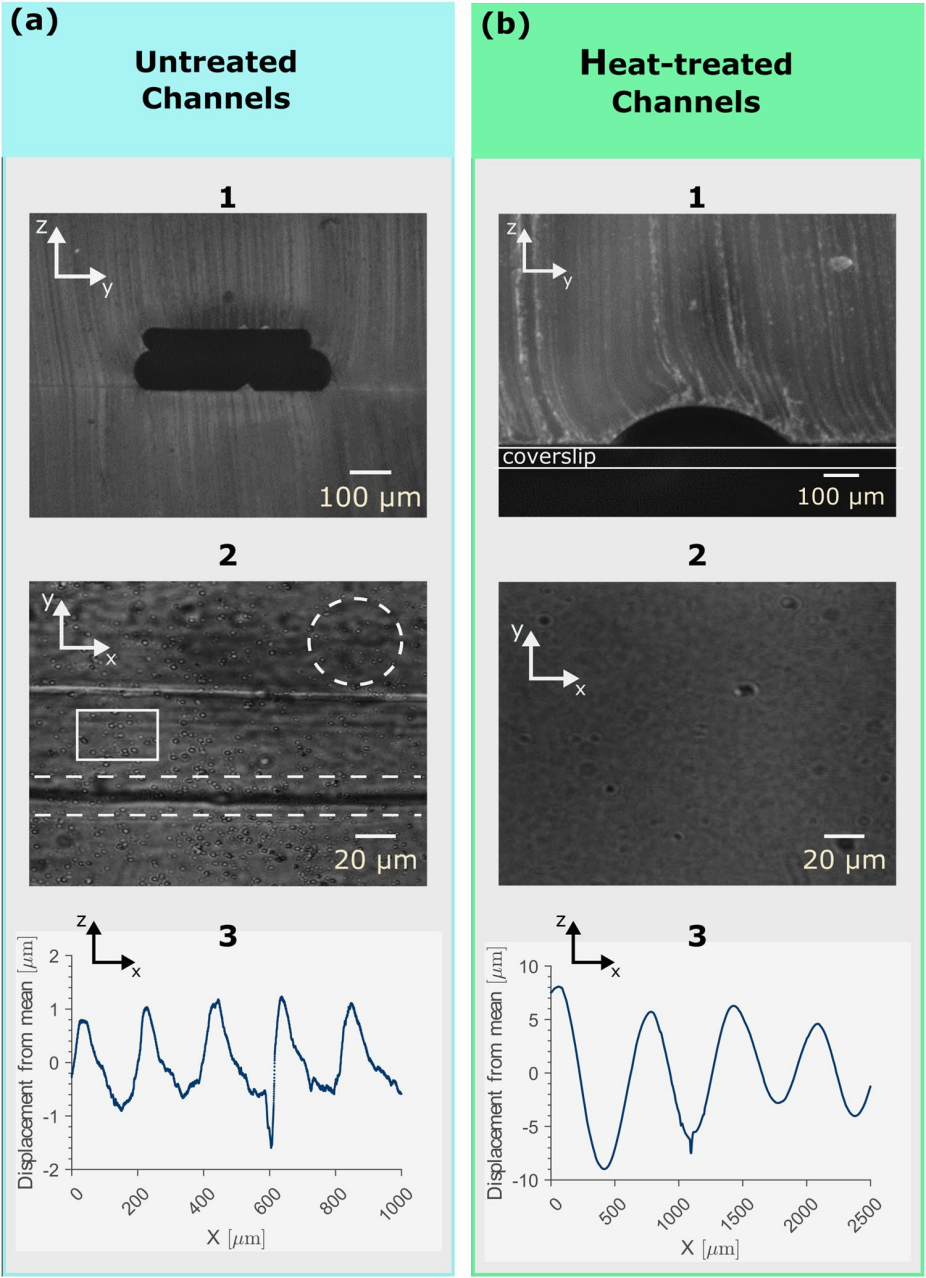
The effect of heating on channel shape, optical transparency and surface roughness. (**a**) Cross section of an untreated channel scaffold entirely embedded in PDMS. (1) We attribute the pancake-stack geometry of the flow channel to the printing properties of our 3D printer. (2) Due to the mentioned irregularities in shape, the optical transparency of the flow chamber is only moderate, since black lines, wavy structures and air bubbles are visible in the image background. (3) Surface roughness of the channel created by an untreated channel scaffold. The discrete movement of the printer nozzle and inhomogeneous plastic extrusion appear as sinusoidal irregularities superimposed by high-frequency noise on the channel surface profile. (**b**) Channel cross section of a PDMS flow chamber with a coverslip as bottom layer. Heating the printed channel scaffold remelts the plastic, resulting in (1) a semi-elliptical channel shape with (2) excellent optical transparency. (3) Surface roughness of the heat-treated channel. Due to heating, only the sinusoidal irregularities from the step-like movement of the printer nozzle remains, improving the optical transparency significantly.

The shape, optical transparency and surface roughness of the molded flow channel strongly depends on the post-printing treatment of the printed channel scaffold. By imaging the cross section of a flow chamber in which the channel scaffold is entirely embedded in PDMS, we observe irregularities in the channel shape, which can be attributed to the printing process. Due to the channel dimensions (height: 80 µm, width: 300 µm) set in Autodesk Inventor, the 3D printer prints two layers of plastic on top of each other. Each layer consists of two filament lines. This procedure results in a pancake-stack geometry of the printout (Fig. 2a panel 1). As a result of this irregular channel shape, the optical transparency of the flow chamber is only moderate, since dents and ditches on its surface can be seen as wavy black objects (Fig. 2a panel 2, inside dashed circle) and dark lines (Fig. 2a panel 2, inside dashed region) in the image background, respectively. Another contribution to the image background arises from small air bubbles which are trapped underneath the channel scaffold during the second molding step. These artifacts appear as spherical objects in the image and reduce the signal-to-noise ratio even more, as they can be mistaken as diffraction patterns of micro-particles (Fig. 2a panel 2, inside rectangle).

In contrast, the heat-threated flow chambers with a coverslip as bottom layer (Fig. 1b) possess a homogeneous shape (Fig. 2b panel 1) with excellent optical transparency (Fig. 2b panel 2). We attribute the smooth channel surface to heating the printed coverslip on a heat plate. The heat re-melts the plastic of the channel scaffold and the surface tension of the plastic reshapes the channel into a regular semi-elliptical cross section while keeping larger structures such as in- and outlets intact. Furthermore, by printing the channel scaffold straight on a coverslip, no air bubbles are embedded at the glass-plastic interface.

To quantify the difference in optical transparency between channels created by untreated and heat-treated channel scaffolds, we probe their surface roughness along the channel on two different length scales, using a profilometer (effective resolution: µm, due to tip radius) and an atomic force microscope (AFM, resolution: nm). Using the profilometer, we find that both channels possess wavy, periodic surface irregularities with a wavelength of several hundreds of micrometers (Fig. 2a panel 3, 2b panel 3, sinusoidal-like part). We attribute these features to the discrete step-like motion of the stepper motors driving the printer nozzle. The amplitude of these irregularities range from 2–20 µm and we find that their frequency depend on the printing speed. High print speed results in a higher frequency whereas lower print speed results in a lower frequency. In this context, we emphasize that these periodic surface irregularities are not only present in the untreated channel scaffold, but also in the heat-treated one. As stated previously, heat-treatment removes only smaller irregularities but leaves bigger structures such as nozzle imprints on the channel scaffold. However, due to their long wavelength we find that these irregularities do not compromise the optical transparency of the flow channel. Additionally, the surface of the channel created by a untreated channel scaffold shows a significant number of small, randomly distributed distortions superimposing the irregularities originating from the regular nozzle movement (Fig. 2a panel 3). They possess amplitudes ranging from sub-micrometer to micrometer and appear as high-frequency noise in the surface profile and originate from an inhomogeneous plastic extrusion during printing. In contrast, heating the channel scaffold after printing reduces the amplitude of these high-frequency irregularities significantly. Consequently, the surface profile becomes smooth (Fig. 2b panel 3), resulting in excellent optical transparency.

To further scrutinize these high-frequency irregularities, we image the surface of the untreated and heat-treated channels using atomic force microscopy. The surface of the untreated channel is distorted with an uneven topology with an arithmetic average surface roughness of *R*_a_ = (47 ± 2) nm, whereas the heat-treated shows a homogeneous surface *R*_a_ = (5 ± 1) nm. Based on these results, we conclude that heat-treatment is an effective procedure to reduce the surface roughness of the printed channel scaffold significantly, resulting in a highly transparent flow channel.

### Heat-Treatment of Channel Scaffold Does Not Lead to Material Evaporation or Shrinkage

To fabricate flow chambers with predictable micro-fluidic properties it is important to know whether the designed channel scaffold in Autodesk Inventor can be realized by the 3D printer or if deviations in physical channel dimensions occur. In this context, we also investigate the effect of heat-treatment on the printed channel scaffold in terms of material shrinkage and evaporation. For these measurements, we design channel scaffolds in Autodesk Inventor with different cross sectional areas *A* and perimeter lengths *L*_p_ (supplementary information: Tables S4 and S5). We design the smallest sample to be 40 µm × 300 µm (A1, height × width) and increase the sample height stepwise by 40 up to channel dimensions of 400 µm × 300 µm (A10) by keeping the channel width constant. Subsequently, we print two identical channel scaffolds of each size and heat-treat one of them. Next, we assess the median cross sectional area *A* and median perimeter length *L*_p_ (see Methods).

**Figure 3:**
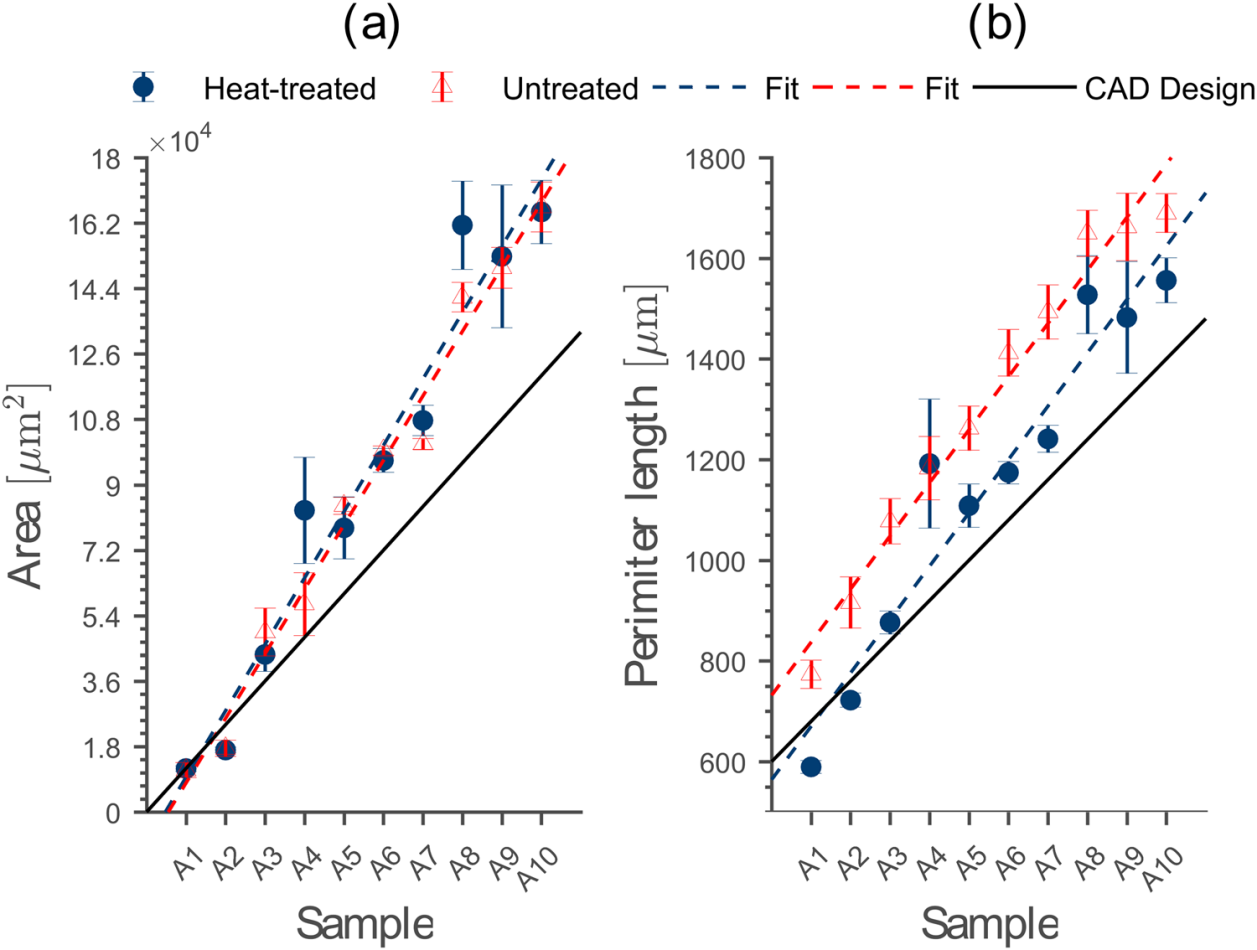
Comparison between printed channel scaffolds with different cross-sectional areas and perimeter lengths and their CAD design. (**a**) The median cross-sectional area *A* of both channel scaffolds show a linear increase with equal slope (untreated: *R*^2^ = 0.96, heat-treated: *R*^2^ = 0.91) with growing channel size. However, both overestimate the cross-sectional area of the CAD design for larger channels. (**b**) Median perimeter length *L*_p_ for the samples from (**a**). Again, untreated (*R*^2^ = 0.97) and heat-treated (*R*^2^ = 0.98) channel scaffolds show a linear increase for different sample sizes. Due to their regular surface, the median perimeter length of the heat-treated channels reproduces the CAD design better than the untreated channel scaffolds. The error bars represent the 95% confidence interval determined from 10 slices each for the 20 individual samples.

We observe a linear increase in the median cross sectional area *A* for both heat-treated and untreated printouts with increasing channel size (Fig. 3a). In average, the cross-sectional area between both printouts differs about 0.6%, indicating that the heating step in the protocol for PDMS flow chambers on a coverslip does not lead to undesirable effects on the channel scaffold such as material shrinkage or evaporation. By comparing the cross-sectional areas of untreated and heat-treated printouts to the ones set in the CAD design (Fig. 3a, black line), we find the smallest deviation for the smallest channel design (Fig. 3a, sample A1). We attribute this observation to the fact that our printer deposits plastic, layer-by-layer, to realize the channel structure. However, each layer can be printed only with a certain accuracy. Consequently, sample A1 (height: 40 µm, width: 300 µm) is closest to the CAD design (deviation: 4% (untreated), 0.4% (heat-treated)), since it consists only of one layer of plastic. By increasing the number of layers (sample A2–A10), deviations in layer height and width start to add up, causing bigger differences between printed cross-sectional area and designed channel scaffold. However, we observe that after printing at least three layers of plastic (sample A3–A10), the deviation between cross-sectional area set in CAD and printout level off around 27.4% (untreated) and 24.4% (heat-treated), respectively. We explain this by a change in the rigidity of the printing environment. The first layer of PVA is printed directly on the rigid glass surface of the build-plate. However, for the second layer the printing environment starts to change due to the softer initial layer of PVA. This transition is completed after printing at least three layers of plastic, resulting in a constant offset between printed cross-sectional area and its CAD design.

In contrast to the median cross sectional area *A*, we find that the perimeter length *L*_p_ of the untreated channel scaffold is in average ≈14% larger than the one for the heat-treated channels (Fig. 3b). This is caused by the heating step during fabrication (Fig. 1b step 5), since it produces a regular channel surface without dents and ditches. Furthermore, the surface tension of the PVA plastic ensures a channel scaffold with minimal perimeter length. Consequently, the heat-treated printout reproduces the CAD design (Fig. 3b, black line) with a median offset of ≈9%, whereas the untreated scaffolds continuously overestimate the perimeter length of CAD design by ≈21%. In agreement with the cross-sectional area, the smallest heat-treated channel scaffolds (sample A1–A3) show the best match in perimeter length compared to their respective CAD designs.

### Height and Width of the Heat-Treated Channel Scaffold is Reproducible and Agrees with the CAD Design

After proving that heat-treating the printed channel scaffold changes only its geometrical shape but does not cause material shrinkage or evaporation, we now probe the repeatability of our 3D printer in printing one channel scaffold multiple times. For that purpose, we print ten copies of sample A1 on a coverslip (40 µm × 300 µm). After heat-treatment, we slice each printout into ten pieces and determine the median channel height *h* and width *w* (see Materials).

**Figure 4:**
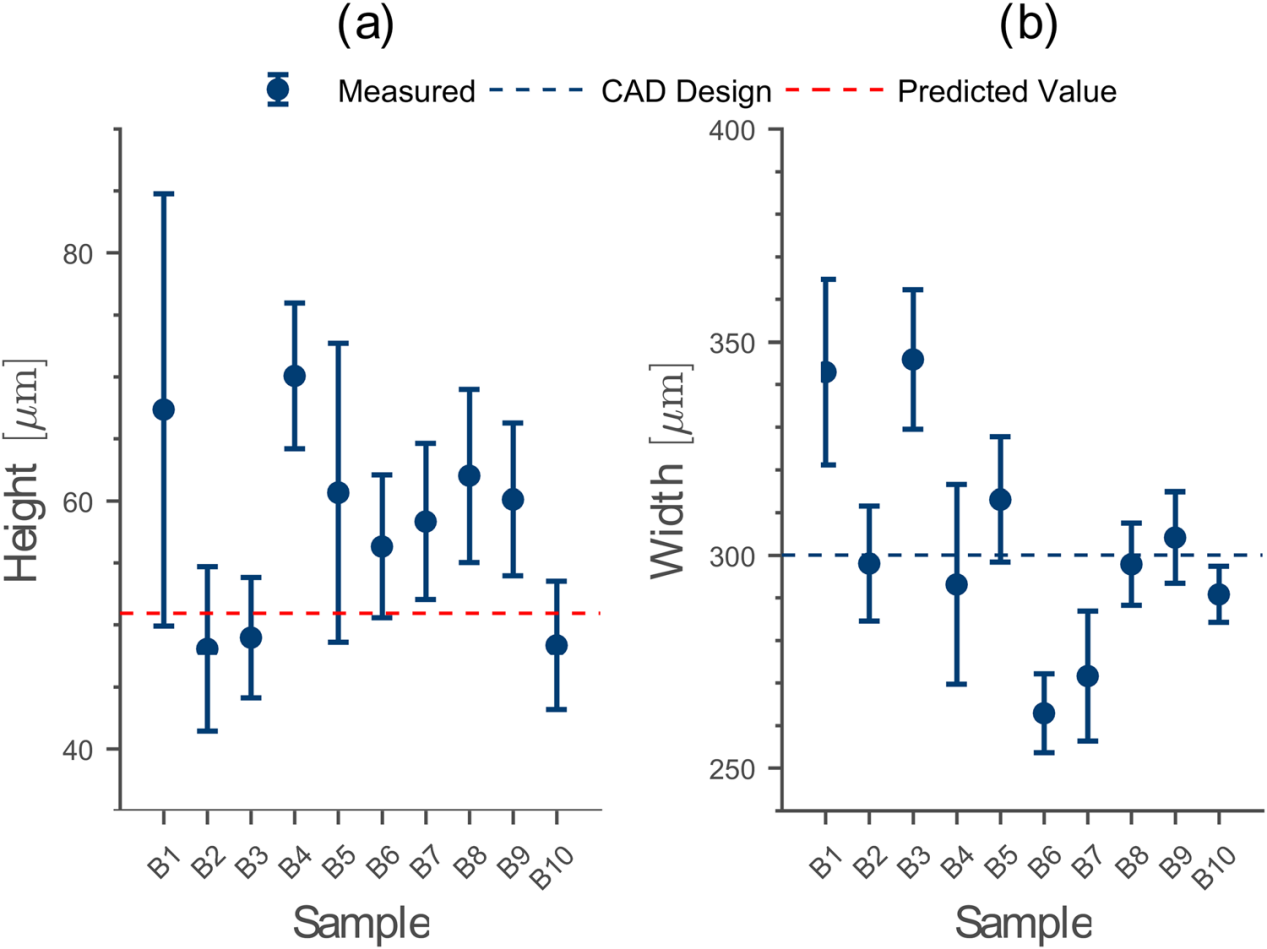
(**a**) Median channel height *h* measured from 10 identical heat-treated channels. The printout height exceeds the predicted height from CAD using equation 1 by ≈14%. (**b**) Median channel width *w* for the same samples from (a). In average the measured channel width coincides with the CAD value within a deviation of 1%. The error bars represent the 95% confidence interval determined from 10 slices each for the 10 individual samples.

We determine the median channel height for all ten samples to *h*_median_ = (59 ± 6) µm (Fig. 4a, supplementary information: Table S6). To compare this value to the height set in CAD, we must consider that heat-treating the channel scaffold transforms its geometry from rectangular into semi-elliptical, without changing the channel width. Thus, we recalculate its height using equation (1) to 51 µm (Fig. 4a, dashed red line), showing that the printout overestimates its design height by 14%. We attribute this deviation to the limited accuracy of our 3D printer in terms of positioning the printer nozzle at the correct height (±5 µm) above the build-plate. This imperfect height positioning can contribute up to 11% to the total deviation of 14%.

By comparing the measured median channel width *w* = (298 ± 11) µm with the value set in CAD (300 µm), we observe a deviation of only 1% (Fig. 4b, supplementary information: Table S6). We explain this excellent agreement by the fact that the printed channel width is mainly determined by two accurately adjustable printer properties: the printer nozzle temperature and the feeding rate of the filament into the 3D printer. By tuning these parameters to their optimum, the agreement in scaffold width can be achieved without changing other printer settings.

### PDMS Flow Channels Produce Predictable Micro-Fluidic Flows

**Figure 5:**
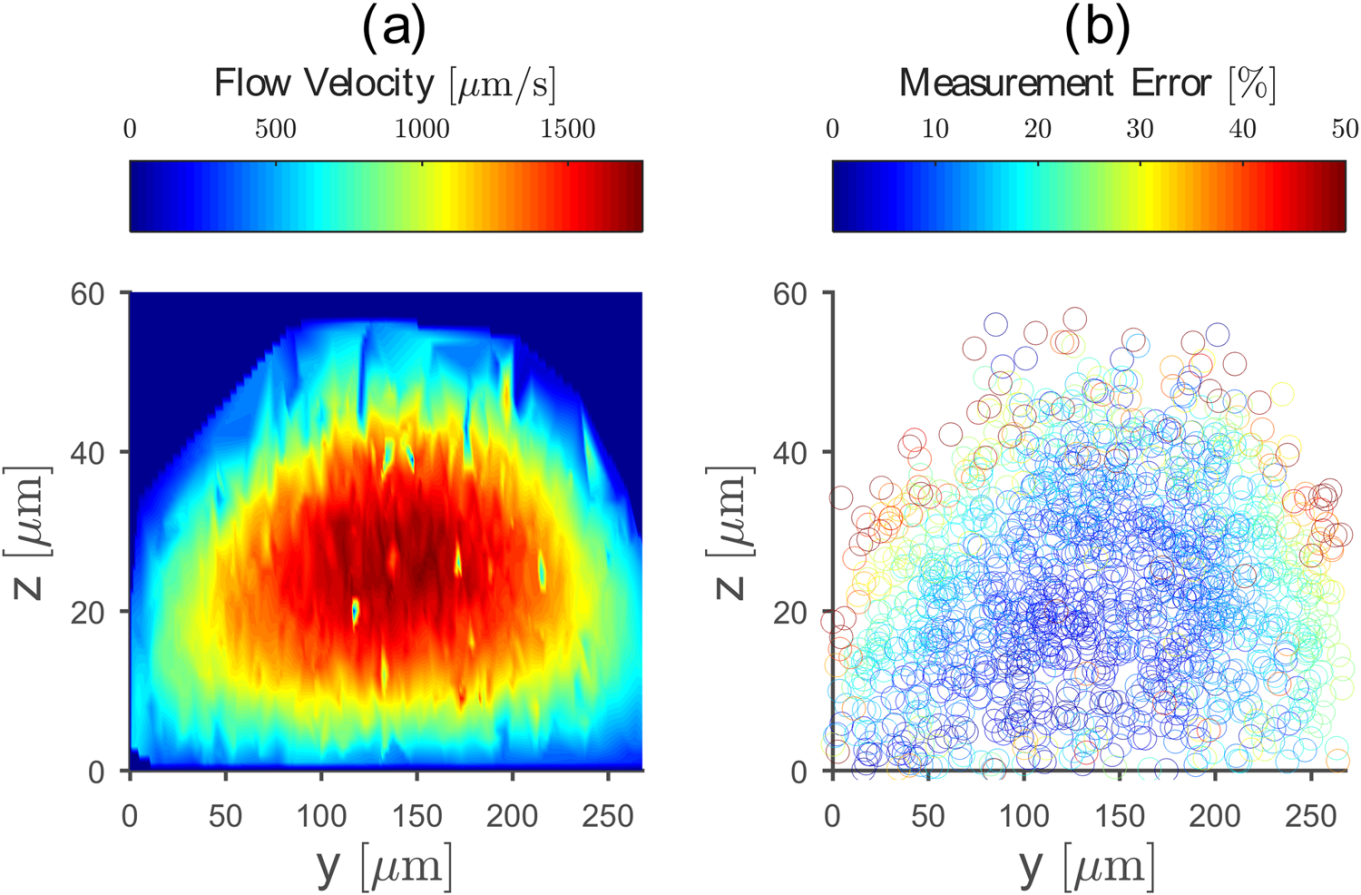
Micro-fluidic flow profile in a semi-elliptical PDMS flow chamber on a coverslip. (**a**) Flow cross section perpendicular to the flow direction. The color code represents the averaged flow velocity at different heights. (**b**) Deviation between measured and theoretical flow profile inside a semi-elliptical channel. We obtain best agreement in the channel middle (blue data points), whereas on the channel walls bigger deviations occur (red data points). The used coordinate system is the same as the one shown in Fig. 2.

After scrutinizing channel properties such as geometrical shape, optical transparency, surface roughness and the channels fabrication repeatability, we now analyze the fluid velocity profile in our flow channels by tracking the velocity of 1 µm particles moving at different heights in the flow using digital holographic microscopy (see Materials). For that purpose, we fabricate a flow chamber on a coverslip (40 µm × 300 µm, CAD height × width) with a semi-elliptical cross section. During the measurement, we create a constant and reproducible volumetric flow rate of (16.7 ± 0.5) nL/s. We position the image plane ≈5 µm below the bottom coverslip and image 1500 particles in a 272 µm × 272 µm (*x*, *y*) field of view. After image analysis using UmUTracker^23^, we calculate the mean speed and mean spatial position (*x*, *y*, *z*) for each particle from their respective trajectory. To visualize the flow profile in a cross section perpendicular to the flow direction, we interpolate the discrete point cloud to obtain a homogeneous surface representation without changing the information content and color-code the data according to the particle speed (Fig. 5a). For the used flow rate, the flow velocity ranges from 39 µm/s to 1766 µm/s for particles close to the channel walls and particles moving in the center of the flow chamber, respectively.

To assess the deviations between measured flow profile and its theoretical prediction, we determine the molded channel dimensions using light microscopy (see Materials). We obtain a channel width *w*_Image_ = (380 ± 1) µm and a channel height *h*_Image_ = (61 ± 7) µm. Using these channel dimensions and a volumetric flow rate of 16.7 nL/s, we calculate the theoretical flow profile in a semi-elliptical flow channel using equation (2) and compute its deviation to the measured one. This comparison is done using the mean position and velocity of each particle trajectory. We find best agreement for the center part of the channel with an average deviation of 5–10% (Fig. 5b, blue data points). However, close to the channel walls and channel bottom, we find a discrepancy of 30–50% between experimental results and theoretical prediction (Fig. 5b, yellow and red data points). Partly, we attribute these deviations to an imprecise particle localization using UmUTracker software. As shown previously, particle tracking and especially height reconstruction for particles close to (near the channel bottom) and far away from (heights > 40 µm) the image plane, becomes error-prone^23^. Since the measured flow profile relies only on the particles coordinates, these tracking errors appear as flow profile deviations in certain channel regions. Further, the deviation between the theoretical and experimental velocity profile can be explained by the fact that the real channel cross section is only approximately semi-elliptical. Thus, in the regions close to the channel walls the relative error becomes large as the theoretical velocity profile tend to zero while the experimental does not. Another contribution to the deviation originates from the channel shape itself. Due to its semi-elliptical shape the channel acts as a weak lens. However, the ratio between refractive indices of the water filled channel and PDMS is only 0.95, thus the deviations caused by the lensing effect are only minor. Despite these discrepancies close to the channel walls, we find excellent agreement in measured and calculated maximum flow velocity (*v*_measured_ = 1766 µm/s, *v*_calculated_ = 1835 µm/s). Consequently, we can estimate the flow profile by measuring its height *h* and width *w*, highlighting the predictable micro-fluidic properties of our flow chamber.

## Conclusions

We present protocols to fabricate micro-fluidic devices by embedding water-soluble 3D printed channel scaffolds in PDMS. Using a 3D CAD design software in combination with a standard commercial available FDM 3D printer, we can realize flow chambers with defined cross sections down to 40 µm × 300 µm. Furthermore, our micro-fluidic devices are transparent, biocompatible, cheap and can be fabricated within 60 min. Since our printer is equipped with a water-soluble, biocompatible filament, no strong solvents or other chemical treatment is necessary to remove the channel scaffold in the hardened PDMS. We characterize our micro-fluidic devices in terms of geometrical shape, optical transparency, surface roughness and repeatability of the printing process. To highlight the reliable fabrication process, we measured the micro-fluidic flow profile in a flow chamber and confirmed our results using theoretical predictions. By providing biocompatible devices with predictable (micro-fluidic) properties, our flow chambers can be used for example to investigate surface-attached bacteria under physiological flow conditions. Furthermore, electronics might be included into the PDMS, making the fabrication protocol also attractive for other lab-on-a-chip applications.

## Methods

### Characterizing Surface Roughness using Profilometry and Atomic Force Microscopy

To measure the surface topography of channel created by an untreated and heat-treated printout, we scanned there surface using a Dektak XT stylus profiler (Bruker, vertical range 524 µm). The profilometer uses a diamond-tipped stylus (radius 12.5 µm, force 3 mg), which moves according to a user-defined scan length and duration (resulting in a resolution in terms of µm/pnt). By defining a width and step length, each scan length is repeated transversely to produce a topography map. For data processing we use the software Vision 64 (Bruker).

To resolve surface irregularities in the sub-micrometer range, we complement our surface analysis with atomic force microscope (AFM) measurements. We operate the AFM in tapping mode using a MMAFMLN Multimode AFM (Veeco Metrology) equipped with a HQ:NSC19/AL BS tip (MikroMasch, radius 8 nm) and a Nanoscope IV controller (Digital Instruments, Veeco Metrology Group). We scan an area of 13.7 µm × 13.7 µm with a rate of 0.5 Hz. To process the data and analyze the surface roughness we use the open source software Gwyddion.

### Measuring the Dimension of the Printed Channel Scaffold

To assess the physical dimensions of our printed channel scaffolds (untreated, heat-treated), we cut them into 10 1 mm thick slices using a razor blade and image each slice under a microscope (Nikon Microphot FX) equipped with a 12 CCD camera (DX 2 HC-VF, Kappa). From these images, we extract the channels cross-sectional area *A* and perimeter length *L*_p_ along with its height *h* and width *w* using the open source software ImageJ^24^.

To compare the height of the heat-treated channel scaffold with its CAD design, we consider that re-melting the printout changes its geometry from rectangular to semi-elliptical. By assuming that the channel width *w* and its cross-sectional area *A* remain unaffected by the heat, the height of the printout is predicted by:

1 $${h}_{{\rm{heat}}-{\rm{treated}}}=\frac{4}{\pi }{h}_{{\rm{CAD}}}\mathrm{.}$$

From equation (1) we note, that due to its semi-elliptical shape the heat-treated channel is ≈27% higher as the height of the corresponding rectangular.

### Flow Velocity Profile Measurements using Digital Holographic Microscopy

To estimate the flow velocity profile in our flow channel, we determine the spatial position of micro-particles using the software UmUTracker, which is based on digital holographic microscopy (DHM). A detailed description of UmUTracker and our experimental setup is published elsewhere^23^.

In brief, we use an Olympus IX70 inverted microscope equipped with a free space objective (Olympus SLMPLN 50x/0.35, ∞/0). The lateral conversion factor of the microscopy system is 160 ± 2 nm/pixel (mean ± standard deviation (Std)). The prepared PDMS cell is mounted onto a piezo stage, which can be positioned in three dimensions over a range of 100 µm with nanometer accuracy using piezo actuators (P-561.3CD, Physik Instrumente). We illuminate the sample from above using a laser (Cobolt 04-01 Series, Calypso 50, *λ* = 491 nm, Cobolt AB, Solna, Sweden) in combination with a rotating ground glass diffuser to remove speckle artifacts in the image background^25^. To increase the image contrast, we position a pinhole (P300S, ST1XY-D, Thorlabs) between light source and flow chamber. Particle trajectories are recorded using a high-speed camera (MotionBLITZ EoSens Cube 7, Mikrotron) operating at 525 fps. After that, we analyze images using the UmUTracker software^23^.

To determine to velocity profile in the flow channel, we use micro-particles (nominal diameter ± Std): (1.040 ± 0.022) µm, Lot No. 15879, Duke Scientific Corp., 4% w/v) suspended in phosphate buffered saline (PBS, pH 7.4). To avoid overlapping diffraction patterns, we optimized the micro-particles concentration by diluting the micro-particles stock solution by 1:200. The diluted micro-particles are pumped through the measurement channel by a syringe pump (Mirus EVO, Celix Ltd, Ireland) equipped with a 100 µL syringe set to deliver a volumetric flow rate of (16.7 ± 0.5) nL/s.

### Theoretical Flow Velocity Profile in a Semi-Elliptical Channel

To validate our flow profile measurements, we compare the experimentally measured velocity profile to the theoretical velocity profile of a semi-elliptical channel^26^:

2 $$u(\xi ,\,\theta )=-\frac{4Q}{\pi wh\bar{u}}\frac{{\sinh }^{2}(\xi ){\sin }^{2}(\theta )}{2{\cosh }^{2}({\xi }_{0})}-\frac{4}{\pi }\sum _{k=1}^{\infty }\frac{\sinh (\xi \mathrm{(2}k-\mathrm{1))}\,\sin (\theta \mathrm{(2}k-\mathrm{1))}}{\mathrm{(2}k-\mathrm{3)(2}k-\mathrm{1)(2}k+\mathrm{1)}\,\sinh \,\mathrm{(2}k-\mathrm{1)}}$$

where

3 $$\bar{u}=(\frac{1}{4}-\frac{2}{{\pi }^{2}}){\tanh }^{2}({\xi }_{0})-\frac{16{\tanh }^{2}({\xi }_{0})}{\pi \,\cosh ({\xi }_{0})}\sum _{k=1}^{\infty }\frac{\sinh \,\mathrm{(2}k{\xi }_{0})}{{\mathrm{(2}k-\mathrm{3)}}^{2}\mathrm{(2}k-\mathrm{1)(2}k+{\mathrm{1)}}^{2}\,\sinh \,\mathrm{((2}k-\mathrm{1)}{\xi }_{0})},$$

and

4 $${\xi }_{0}=\,\tanh (\frac{w}{2h})\mathrm{.}$$

*ξ* and *θ* are the elliptic cylindrical coordinates, *w* is the channel width, *h* is the channel height and *Q* is the volumetric flow rate.

### Data availability

The datasets generated during and/or analysed during the current study are available at 10.6084/m9.figshare.5182597.v2.

## Electronic supplementary material

Supporting Information
