# Supplementary material for: 3D printed water-soluble scaffolds for rapid production of PDMS micro-fluidic flow chambers
Source: Sci Rep. 2018 Feb 20;8:3372. doi: 10.1038/s41598-018-21638-w (PMC5820269; doi:10.1038/s41598-018-21638-w)
Supplement: Supplementary file 1 — Supporting Information [file 41598_2018_21638_MOESM1_ESM.pdf]

3D printed water-soluble scaffolds for rapid production of PDMS micro-fluidic flow chambers

Tobias Dahlberg[1], Tim Stangner[1], Hanqing Zhang[1], Krister Wiklund[1], Petter Lundberg[1], Ludvig Edman[1], and Magnus Andersson[1,*]

[1]Department of Physics, Umeå University, 901 87 Umeå, Sweden
[*]magnus.andersson@umu.se

Supporting information

Electronic Supplementary Information (ESI) are available at:
https://figshare.com/articles/Supplementary_Information/5182597.

Slicing Software Cura (version 2.6.1) and Ultimaker 2+ Material Settings

Table S1. A list of the critical slicing software (Cura) settings, printer material and their respective values.

Cura (Version 2.6.1) Settings	Value	Ultimaker 2+ Material Settings	Value
Layer Height	0.04 mm	Printing Temperature	190 °C
Initial Layer Height	0.04 mm	Bed Temperature	60 °C
Line Width	0.295 mm	Flow	45 %
Infill Line Width	0.18 mm	Diameter	3.1 mm
Wall Thickness	0.295 mm		
Top/Bottom Thickness	0.04 mm		
Infill Density	100 %		
Print Speed	15 mm/s		
Top/bottom Speed	15 mm/s		
Initial Layer Speed	15 mm/s		
Print Jerk	5 mm/s		
Print Acceleration	200 mm/s^2		
Fan Speed	100 %		
Initial Fan Speed	0 %		
Regular Fan speed at Height	0.2 mm		

Channel Scaffolds Cover a Broad Width and Height Range in Agreement with their CAD Design

To prove that our proposed fabrication protocols can produce flow chambers width custom-made height and width, we design channel scaffolds in Autodesk Inventor by varying these two parameters. In this context, we also investigate if deviations between the physical channel dimensions set in Autodesk Inventor and the printout occur. First, we design channel scaffolds with fixed height ($h = 100\,\mu$m) and vary their width from $w \in [300,1000]\mu$m by increasing the channel width stepwise by 70 µm (sample C1-C11). Second, we repeat the design step but keep the channel width constant ($w = 300\,\mu$m) and vary the height h from 40–400 µm in discrete 40 µm steps (Sample D1-D10). Third, we print all samples, slice each printout into 10 slices and determine their median width and median height (see Methods). Please note, the channel scaffolds use in these measurements are not heat-treated.

For samples C1-C11 we determine the constant median height to $h = (94 \pm 7)\,\mu$m, reproducing its CAD design value with a median deviation of 6 % (Fig. S1a, Table S3). Furthermore, we observe a linear increase in the median width, in agreement with its CAD design (Fig. S1a, median deviation: 4 %). By keeping the width of the printout constant and changing its height, we observe the following (Fig. S1b, Table S2): (1) The sample height increases linear and agrees with its CAD design value (median deviation: 6 %). (2) The medium width (w=(395 ± 15) µm) varies slightly for sample D1-D10, but overestimates its CAD value by almost 100 µm.

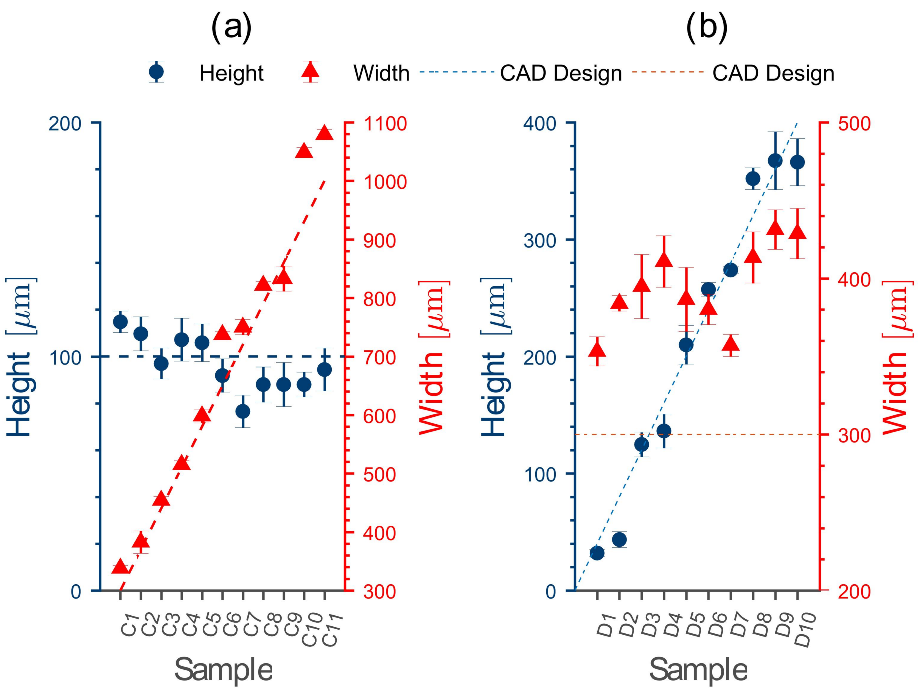

Figure S1. Comparison between printed channel scaffolds with varying width and height and their CAD design. (a) Channel scaffolds with fixed height (full blue spheres) and varying width (open red triangles). The median height stays constant for all samples C1-C11 and agrees with its CAD design value (blue dashed line). The median width increases linearly ($R^2 = 0.97$) and reproduces its CAD design value (red dashed line). (b) Channel scaffolds with fixed width (open red triangles) and varying height (full blue spheres). The median width varies slightly for all samples and overestimates its CAD design value (red dashed line). The median height increases linearly ($R^2 = 0.97$) and reproduces its CAD design value (blue dashed line). The error bars represent the 95 % confidence interval determined from 10 slices each for individual sample.

Table S2. Comparison between the width of the channel scaffold ($w_{exp} \pm$ Std) at constant height according to dimensions set in Autodesk Inventor (CAD), and their respective relative deviations from each other.

Sample	h_{CAD} [µm]	w_{CAD} [µm]	h_{exp} [µm]	w_{exp} [µm]	$\Delta\left(\frac{h_{CAD}}{h_{exp}}\right)$ [%]	$\Delta\left(\frac{w_{CAD}}{w_{exp}}\right)$ [%]
C1	100	300	115 ± 5	338 ± 5	15	13
C2	100	370	110 ± 7	383 ± 19	10	3
C3	100	440	97 ± 7	454 ± 7	3	3
C4	100	510	107 ± 9	515 ± 7	7	1
C5	100	580	106 ± 8	598 ± 12	6	3
C6	100	650	92 ± 7	737 ± 5	8	13
C7	100	720	77 ± 7	750 ± 13	23	4
C8	100	790	88 ± 8	821 ± 6	12	4
C9	100	860	88 ± 9	833 ± 21	12	3
C10	100	930	88 ± 5	1048 ± 8	12	13
C11	100	1000	94 ± 9	1079 ± 9	6	8
Median					10	4

Table S3. Comparison between the height of the channel scaffold ($h_{exp} \pm$ Std) at constant width according to dimensions set in Autodesk Inventor (CAD), and their respective relative deviations from each other.

Sample	h_{CAD} [µm]	w_{CAD} [µm]	h_{exp} [µm]	w_{exp} [µm]	$\Delta\left(\frac{h_{CAD}}{h_{exp}}\right)$ [%]	$\Delta\left(\frac{w_{CAD}}{w_{exp}}\right)$ [%]
D1	40	300	32 ± 3	353 ± 9	20	18
D2	80	300	44 ± 7	384 ± 5	46	28
D3	120	300	125 ± 11	395 ± 21	-4	32
D4	160	300	136 ± 15	411 ± 17	15	37
D5	200	300	210 ± 17	386 ± 21	-5	29
D6	240	300	257 ± 6	380 ± 10	-7	27
D7	280	300	274 ± 3	357 ± 7	2	19
D8	320	300	352 ± 9	413 ± 16	-10	38
D9	360	300	367 ± 25	431 ± 13	-2	44
D10	400	300	366 ± 20	429 ± 16	8	43
Median					8	30

Measurement Data for Channel Cross Section and Perimeter Length, Channel Height and Width

Table S4. Comparison between the cross-sectional area of the channels ($A \pm \mathrm{Std}$) according to dimensions set in Autodesk Inventor (CAD), measured for the untreated and heat-treated channel scaffolds and their respective relative deviations from each other.

Sample	A_{CAD} [10^4 µm^2]	$A_{\mathrm{untreated}}$ [10^4 µm^2]	$A_{\mathrm{heat\text{-}treated}}$ [10^4 µm^2]	$\Delta\left(\frac{A_{\mathrm{CAD}}}{A_{\mathrm{untreated}}}\right)$ [%]	$\Delta\left(\frac{A_{\mathrm{CAD}}}{A_{\mathrm{heat\text{-}treated}}}\right)$ [%]	$\Delta\left(\frac{A_{\mathrm{untreated}}}{A_{\mathrm{heat\text{-}treated}}}\right)$ [%]
A1	1.2	1.2 ± 0.2	1.2 ± 0.1	-4.0	-0.37	-3.7
A2	2.4	1.8 ± 0.2	1.7 ± 0.1	-37.1	-40.8	2.7
A3	3.6	5.0 ± 0.7	4.3 ± 0.5	27.3	16.9	12.5
A4	4.8	5.7 ± 0.9	8.3 ± 1.4	16.1	42.2	-45.1
A5	6.0	8.4 ± 0.2	7.8 ± 0.9	28.8	23.2	7.2
A6	7.2	9.9 ± 0.1	9.7 ± 0.3	27.6	25.5	2.7
A7	8.4	10.1 ± 0.1	10.8 ± 0.4	17.0	22.0	-6.3
A8	9.6	14.2 ± 0.4	16.1 ± 1.2	32.2	40.5	-14.0
A9	10.8	15.0 ± 0.6	15.3 ± 2.0	27.9	29.3	-2.1
A10	12.0	16.6 ± 0.7	16.5 ± 0.7	27.9	27.3	0.8
Median				27.4	24.4	0.6

Table S5. Comparison between the perimeter length of the channel ($L_p \pm$ Std) according to dimensions set in Autodesk Inventor (CAD), measured for the untreated and heat-treated channel scaffolds and their respective relative deviations from each other.

Sample	$L_{p,\, CAD}$ [10^2 µm]	$L_{p,\, untreated}$ [10^2 µm]	$L_{p,\, heat\text{-}treated}$ [10^2 µm]	$\Delta\left(\frac{L_{p,\, CAD}}{L_{p,\, untreated}}\right)$ [%]	$\Delta\left(\frac{L_{p,\, CAD}}{L_{p,\, heat\text{-}treated}}\right)$ [%]	$\Delta\left(\frac{L_{p,\, heat\text{-}treated}}{L_{p,\, untreated}}\right)$ [%]
A1	6.8	7.7 ± 0.3	5.9 ± 0.1	12.1	-15.3	23.8
A2	7.6	9.2 ± 0.5	7.2 ± 0.1	17.0	-5.2	21.2
A3	8.4	10.8 ± 0.4	8.8 ± 0.2	22.0	4.2	18.6
A4	9.2	11.8 ± 0.6	11.9 ± 1.3	22.2	22.8	-0.8
A5	10.0	12.6 ± 0.4	11.1 ± 0.4	20.8	9.8	12.2
A6	10.8	14.1 ± 0.5	11.8 ± 0.2	23.5	8.0	16.8
A7	11.6	14.9 ± 0.5	12.4 ± 0.3	22.3	6.5	16.9
A8	12.4	16.5 ± 0.5	15.3 ± 0.8	24.8	18.8	7.4
A9	13.2	16.6 ± 0.7	14.8 ± 1.1	20.6	11.0	10.8
A10	14.0	16.9 ± 0.4	15.6 ± 0.4	17.1	10.0	7.9
Median				21.4	8.9	14.5

Table S6. Measured median channel height ($h \pm$ Std) and width ($w \pm$ Std) of ten identical heat-treated printouts and their deviation to the CAD design. The dimensions in CAD are set to 40 µm × 300 µm (height x width). However, heat-treatment of the channel scaffold changes its geometrical shape and its height must be recalculated using equation (**??**), resulting in a channel scaffold of 51 µm × 300 µm (height x width).

Sample	$h_{heat\text{-}treated}$ [µm]	$w_{heat\text{-}treated}$ [µm]	$\Delta\left(\frac{h_{CAD}}{h_{heat\text{-}treated}}\right)$ [%]	$\Delta\left(\frac{w_{CAD}}{w_{heat\text{-}treated}}\right)$ [%]
B1	67 ± 17	343 ± 20	24	-14
B2	48 ± 7	298 ± 14	-6	1
B3	49 ± 5	346 ± 16	-4	-15
B4	70 ± 6	293 ± 23	27	2
B5	61 ± 12	313 ± 15	16	-4
B6	56 ± 7	263 ± 9	9	12
B7	58 ± 6	272 ± 15	12	9
B8	62 ± 7	298 ± 10	18	1
B9	60 ± 6	304 ± 11	15	-1
B10	48 ± 5	291 ± 7	-6	3
Median	59 ± 6	298 ± 11	14	1
